# Optical absorption signature of a self-assembled dye monolayer on graphene

**DOI:** 10.3762/bjnano.7.78

**Published:** 2016-06-14

**Authors:** Tessnim Sghaier, Sylvain Le Liepvre, Céline Fiorini, Ludovic Douillard, Fabrice Charra

**Affiliations:** 1 SPEC, CEA, CNRS, Université Paris Saclay, CEA/Saclay 91191 Gif-sur-Yvette Cedex, France

**Keywords:** absorption spectroscopy, dye aggregates, graphene, molecular monolayer, optical spectroscopy, perylene-3,4,9,10-tetracarboxylic-3,4,9,10-diimide (PTCDI), scanning tunnelling microscopy, self-assembly, self-organization

## Abstract

A well-organized monolayer of alkylated perylene-3,4,9,10-tetracarboxylic-3,4,9,10-diimide (PTCDI) has been formed onto CVD graphene transferred on a transparent substrate. Its structure has been probed by scanning tunnelling microscopy and its optical properties by polarized transmission spectroscopy at varying incidence. The results show that the transition dipoles of adsorbed PTCDI are all oriented parallel to the substrate. The maximum absorption is consistent with the measured surface density of molecules and their absorption cross section. The spectrum presents mainly a large red-shift of the absorption line compared with the free molecules dispersed in solution, whereas the relative strengths of the vibronic structures are preserved. These changes are attributed to non-resonant interactions with the graphene layer and the neighbouring molecules.

## Introduction

Close-packed assemblies of dye molecules exhibit drastically altered photonic properties as compared with the isolated or diluted species [1]. These changes find their origin in near-field optical interactions between the constituent molecules, as early predicted by McRay and Kasha [2]. A well-known example is the spectral shift induced by the self-association of cyanine dyes in solution [3]. Depending on the aggregation pattern of the dyes, either bathochromically shifted *J*-bands or hypsochromically shifted *H*-bands are formed, corresponding to collectively excited states and energy bands of delocalized excitons [4–5]. The giant transition dipole moments associated with such excitations result in enhanced optical interactions, e.g., with plasmon resonators in which case a strong-coupling regime can then be reached [6–7]. These collective excitations can also lead to remarkable light emission processes such as superradiance [8]. Interactions between a dye and its surroundings at the molecular scale may also induce drastic changes in its photonic properties. Structural planarization of the adsorbed molecules [9], or the immersion inside a polarizable medium [10] can induce uniform bathochromic shifts of the vibronic peaks constituting the absorption spectrum. Finally, when distances between π-conjugated systems are small enough to permit electron tunnelling, quantum effects can also come into the play, at the origin, for instance, of new intermolecular charge-transfer absorption peaks [11]. Consequently, the fine tuning of molecular-scale organization of condensed dye assemblies appears critical for the control of photonic properties of matter and possibly for the generation of original photonic processes.

An atomically precise positioning of self-associated molecular dyes can be achieved either in vacuum or at the solution–substrate interface by self-assembly techniques. In particular, perylene-3,4,9,10-tetracarboxylic-3,4,9,10-diimide (PTCDI) and its sibling molecule perylene-3,4,9,10-tetracarboxylic dianhydride (PTCDA), have become archetypes for photonic applications of dyes [12], for self-organized adsorption on various atomically flat surfaces [13], and for their combination. Indeed, optical differential reflectance spectroscopy [14], photoluminescence, or Raman diffraction studies have evidenced optical responses attributed to strong interactions of PTCDA with metal [15–16] or semiconductor [17] substrates and between neighbouring molecules when deposited on a dielectric substrate [18–19] or in multilayer structures [20]. The optical effects of interactions between close-packed PTCDA molecules deposited on epitaxial graphene have also been observed [21]. In turn, self-assembly of adsorbed conjugated molecules can influence the electronic properties of its substrate. Such a non-covalent functionalization is especially suitable in the case of graphene because of its “surface only” nature [22–23] and has been applied to tailor its band structure [24] or its work function [25–26] with a monolayer of PTCDI and similar molecules, which can be laterally patterned [27] or even manipulated at the single-molecule level [28].

Beyond H-bond-steered organizations [29], a high level of control of the self-assembly geometry is possible by exploiting the interactions between alkyl side chains and the surface of highly oriented pyrolytic graphite (HOPG) [30]. Based on these principles, it has been possible to design molecular building blocks that arrange spontaneously according to various predetermined patterns [31]. These techniques can be extended to monolayer CVD graphene as a substrate [32], which offers optical transparency when transferred from its native CVD substrate –usually copper– onto a transparent one such as quartz or polyethylene terephthalate (PET). This offers opportunities for advanced optical characterizations in a transmission geometry, such as polarized variable-incidence transmission spectroscopy. In addition, the electrical conductivity of a CVD graphene monolayer is sufficiently high to apply scanning tunnelling microscopy (STM) and thus determine the structural data of the molecular assembly with atomic-scale accuracy. Through the combination of optical characterizations and structural control of dye assemblies on CVD graphene, detailed studies about the influence of the dye organization on photonic properties become feasible.

In this paper, we explore the changes in the optical transmission spectrum of an alkylated derivative of PTCDI upon its self-assembly onto a CVD graphene monolayer, and analyse the results based on STM data taken on the very same sample.

## Results

### Scanning tunnelling microscopy

**Figure 1:**
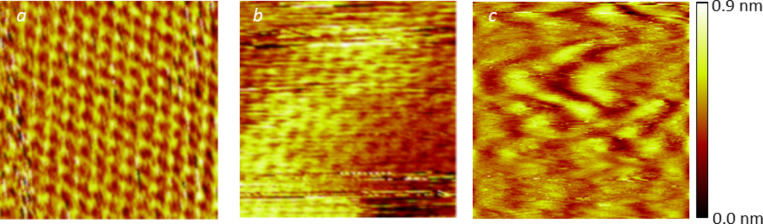
Structural characterization of the substrates. STM images (2.3 × 2.3 nm^2^) of a HOPG surface (a) and CVD monolayer graphene transferred onto a fused quartz plate (b: 2.3 × 2.3 nm^2^ and c: 50 × 50 nm^2^). The images were acquired under air atmosphere, in the height (constant current) mode. The setpoint current was *I*_S_ = 100 pA, and the bias was *V*_T_ = 200 mV. The height scale is also shown for graphene on quartz (c).

The self-assembly was probed on two graphitic substrates, highly oriented pyrolytic graphite (HOPG) and monolayers of CVD graphene transferred either onto fused silica (“optical quartz”) or PET. The resolution of carbon atoms is easily obtained on both systems. On CVD graphene samples, an additional moderate roughness is observed, which is attributed to the substrate (Figure 1a,b). For example, in the case of graphene transferred onto a fused silica plate, this roughness attains 0.5 nm over distances of approximately 50 nm (Figure 1c).

**Figure 2:**
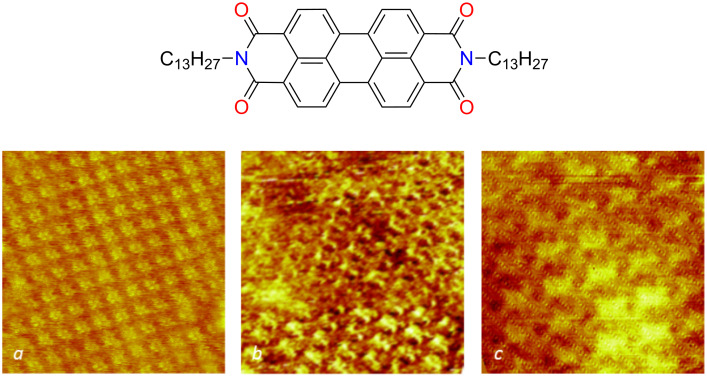
Structural characterization of the self-assembled PTCDI monolayers. Molecular formula of PTCDI-C13 and STM images of self-assembled monolayers on HOPG (a: 14 × 14 nm^2^) as-grown CVD monolayer graphene on copper foil (b: 11 × 11 nm^2^) and CVD graphene monolayer transferred onto a PET plate (c: 8 × 8 nm^2^). The typical current setpoint and sample bias were 10 pA and 800 mV, respectively. The images were acquired at the interface between the substrate and a ca. 10^−5^ M solution in phenyloctane at room temperature.

The PTCDI molecule has become a paradigm both as a self-assembly tecton and as a dye. For the present study, we chose an alkylated form of this dye, *N*,*N*′-ditridecylperylene-3,4,9,10-tetracarboxylic diimide (PTCDI-C13), in order to take advantage of the interactions between *n*-alkyl chains and graphitic substrates for forming a spontaneously self-assembled monolayer at the interface between the solution and graphene. The monolayer structures have been studied by STM at the solution–substrate interface. Intramolecular resolution is possible both with HOPG and graphene as substrates (Figure 2). As expected from the atomically flat surface of HOPG, this substrate produces the largest domains. It permits an accurate determination of the lattice parameters, which correspond to a surface density of 0.45 molecules per nm^2^ and a distance between closest neighbours of ca. 1.4 nm. The network obtained on CVD graphene is compatible with that obtained on HOPG, with one molecule per unit cell. The various domains have a finite number of lattice orientations, indicating an epitaxial relationship with the graphitic lattice. These results are fully consistent with the expected formation of a self-assembled monolayer in which the molecules are lying flat on the substrate, with adsorbed *n*-alkyl chains aligned on the C-atom lattice. By randomly inspecting various regions of drop-cast samples, it appears that a nearly complete coverage (about 80–90%) is obtained whereas the droplet spread on the sample contained the exact quantity of molecules needed to form a monolayer (see Experimental section). The quantitative formation of multilayers can thus be ruled out.

### Transmission spectra

**Figure 3:**
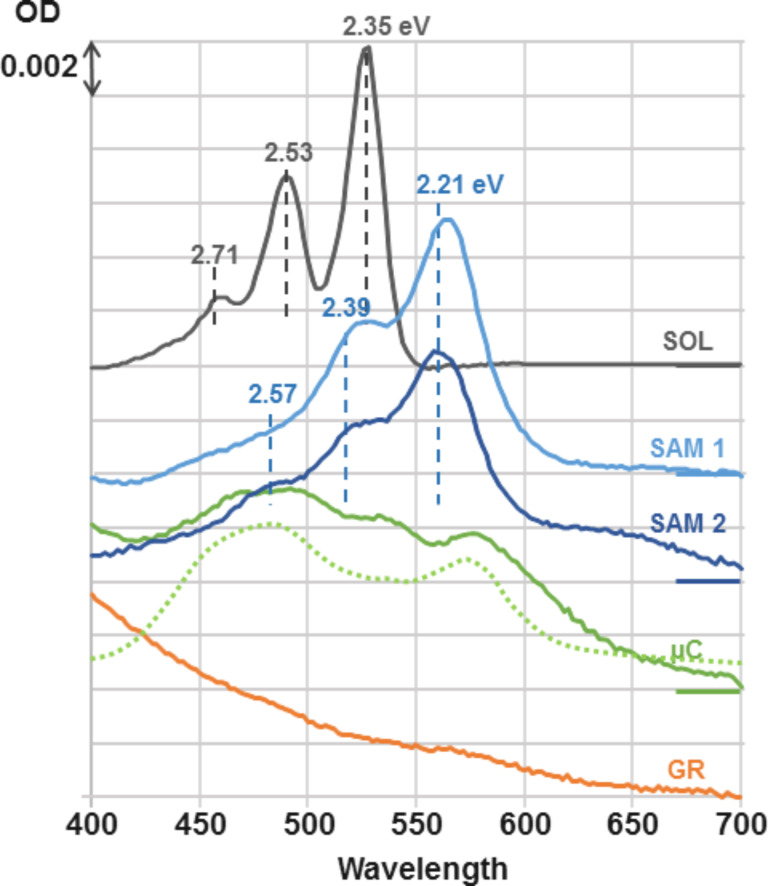
Normal incidence transmission spectra *T*, expressed as an optical density DO = −log(*T*/*T*_0_). SOL: a 10^−6^ M solution of PTCDI in toluene, cell thickness: 2 mm (black curve). SAM1 and SAM2: two self-organized PTCDI-C13 monolayers on graphite, formed following two methods: drop casting (light blue, SAM1) or dip coating (dark blue, SAM2). µC: 10 equivalent monolayers of PTCDI-C13 deposited by solvent evaporation on a fused-quartz substrate, that is without CVD graphene (green curve) and PTCDI-C13 microcrystalline thin film (dotted green curve) reproduced after Mizuguchi et al. [36] and rescaled for easier comparison. GR: monolayer CVD graphene transferred onto a fused-quartz plate (orange curve). All spectra are referenced (*T*_0_) to their corresponding naked substrate (GR for SAM1 and SAM2, quartz plate for µC and GR) or pure solvent (SOL). The energies of the 0–0, 0–1 and 0–2 vibronic peaks are indicated for solution and monolayer spectra.

The solution spectrum of PTCDI-C13 is reported in Figure 3 (labelled “SOL”). It presents the typical vibronic structure of a π–π* transition, with an energy difference of 0.18 eV (ca. 1450 cm^−1^) between 0–0, 0–1 and 0–2 sub-bands which is a characteristic of the π-conjugated systems as present in the PTCDI core [33]. The main peak is the 0–0 at 2.35 eV and corresponds to an absorption cross section σ_SOL_ = 3.3 × 10^−16^ cm^2^ consistent with the molar attenuation coefficient reported in the literature for alkylated PTCDI [34]. Quantum chemical calculations have shown that the transition dipole moment corresponding to the π–π* transition is aligned along the N–N′-axis [33]. The difference transmission spectra between self-assembled monolayer on a substrate of monolayer CVD graphene transferred onto fused quartz are shown in Figure 3. Two types of depositions are reported: (i) drop casting of a droplet of a toluene solution containing the exact amount of molecules needed for a coverage of 0.45 molecule per nm^2^ (SAM 1) and (ii) dip-coating in a ca. 10^−5^ M solution in toluene followed by rinsing in toluene and ethanol (SAM 2). The transmission spectra of SAM1 and SAM2 are nearly identical and correspond to a rigid bathochromic shift of 0.14 eV (1130 cm^−1^) of the whole vibronic system. This quantitative similarity further supports the homogeneous formation of one monolayer by dip coating, as was shown in the case of drop casting. At the maximum of the 0–0 absorption peak, shifted to 2.21 eV, the relative transmission Δ*T*/*T* is ca. 2.1% (SAM1) and ca. 1.9% (SAM2). For an absorbing monolayer deposited on a lossless dielectric substrate, the relative transmission depends only on the absorptive part of the molecular optical response [35]. Given the molecule surface density of 0.45 molecule per nm^2^ measured by STM, the molecular absorption cross section can be thus evaluated to σ_ML_ = 4.5 × 10^−16^ cm^2^. By assuming an in-plane orientation of the N–N′-axis, which gives a factor of 3/2 corresponding to a 2D orientational averaging of the transition dipole moments instead of the 3D averaging in solution, the above value is fully consistent with that obtained in solution (3.3 × 10^−16^ cm²). Notice that accounting for the Lorentz local field correction to σ_SOL_, [(*n*^2^ + 1)/3]^2^/*n* with *n* being the index of refraction of toluene, and for the substrate-index correction to σ_ML_, (*n* + 1)/2 with *n* being the index of the substrate [35], does not change this conclusion (ca. 1.33 and ca. 1.25, respectively). Micro-spectroscopy sampling of the sample, averaged over a spot of about 3µm in diameter have shown a high homogeneity of the absorption in the range of millimetres.

Remarkably, in the absence of graphene coverage on the fused-quartz substrate prior to PTCDI-C13 deposition, completely different transmission spectra are observed. Actually, no measurable absorption is recorded after using the dip coating technique and the spectrum observed for drop casting is very similar to the one reported in the literature for PTCDI microcrystalline films [36] (µC, solid and dotted lines). Moreover, micro-spectroscopy has shown a high inhomogeneity of the absorption strength, whereas the measured spectra acquired at different places remain homothetic.

**Figure 4:**
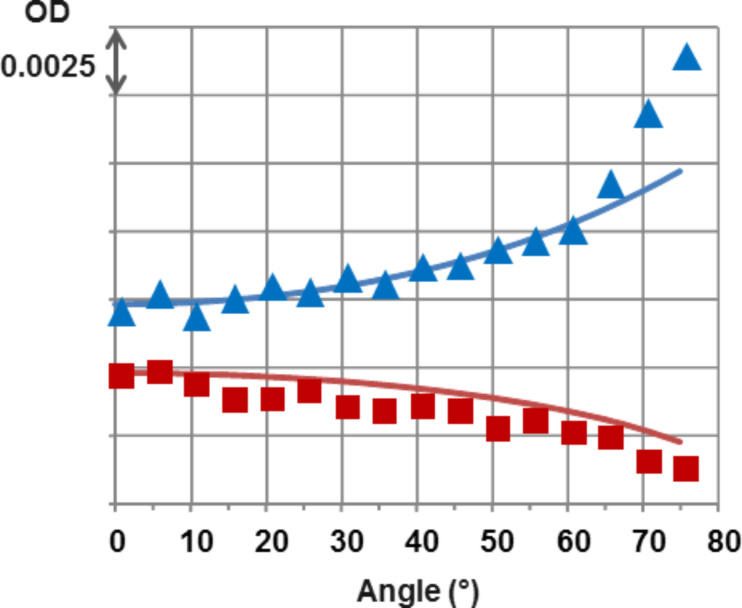
Optical signature of orientations of self-organized PTCDI-C13. Variable-incidence polarized-transmission analysis of the contribution of the self-assembled molecular monolayer to the absorption (blue triangle: TE, red squares: TM). The optical density at the absorption maximum (λ = 561 nm), as obtained from a fit of the absorption line of the molecule, is plotted. The continuous lines represent the TE and TM theoretical absorption variations [37] considering molecular transition dipole moments lying flat on graphene. For randomly-oriented transition dipole moments, both TE and TM should increase with incidence following the blue line.

Finally, we have measured the dependence on the incidence angle of the PTCDI absorption spectral feature in the polarized absorption for SAM1 and SAM2 samples (Figure 4). Whereas the absorption increases with incidence for TE polarization (blue triangles in Figure 4), it decreases monotonically with increasing incidence for TM polarization (red squares). In both cases, the molecule spectrum changes homothetically, that is preserving the balance between vibronic peaks. These observation confirm that the orientations of the transition dipole moments of the molecule are parallel to the substrate surface (graphene layer), as shown by the theoretical model [37] (continuous lines). This effect is even visible to the naked eye looking through a tilted plate through a polarizer. Notice that, for a thin film with random 3D molecule orientation, both TE and TM polarizations should exhibit an increased absorption at grazing incidence.

The quantitative spectral analysis shows that the characteristic optical absorptions of drop-cast (SAM1) and dip-coated (SAM2) samples result from a homogeneous assembly of flat lying molecules with a surface density equivalent to one monolayer. Together with STM observations, this permits to unambiguously assign the spectral features observed at 2.21, 2.35 and 2.57 eV to the absorption of the PTCDI-C13 monolayer self-assembled onto the graphene substrate with a planar geometry of the molecules. The most striking feature of the self-assembled monolayer spectra is the uniform red-shift of the whole vibronic spectral line, which results from the self-assembly. This shift is not accompanied by an important blurring of the spectral structures, consistent with the high homogeneity of molecular organization and environments obtained through the atomically precise self-assembly process on graphene. The energy difference between 0–0, 0–1 and 0–2 sub-bands is preserved, at 0.18 eV. This value is characteristic of the π-conjugated C–C double bond vibration and shows that the absorption remains dominated by the π–π* transition.

2D ordered aggregation of similar molecules on metals have been reported to induced drastic changes in optical spectra, attributed to new electronic transitions [16]. Less pronounced rigid spectral shifts have been reported previously for molecules deposited on crystalline dielectrics such as hexagonal boron nitride (h-BN) [9,19]. The cited possible origins of such shifts are optical interactions between molecules or between molecules and the substrate and the deformation of molecules induced by van der Waals interactions between molecules and substrate. Intermolecular optical interactions are a consequence of the local electric field resulting from the induced dipoles of molecules at neighbouring sites [38]. For an assembly of molecules in free space, this relative change is


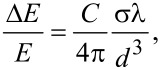


where σ is the absorption cross-section (3.3 × 10^−16^ cm^2^), λ the excitation wavelength in vacuum (ca. 0.5 µm), *d* the distance between molecules (1.4 nm), and *C* a geometrical factor typically of the order of unity. This factor is about 50%, which would be more than sufficient to explain the large shift observed here. However, such resonant interactions should also lead to a concentration of the oscillator strength on the 0–0 vibronic transition [39]. Yet, in the present case, a fit of the absorption line accounting for the spectral broadening of the vibronic structures gives an increase of the 0–0 to 0–1 ratio of only about 20%, which is not consistent with the expected exciton delocalization. A substrate-induced planarization was invoked for hydrogen-bonded porphyrins on h-BN [9]. However, the PTCDI molecule already present a rigid intrinsically planar covalent structure and substrate-induced planarization cannot explain the even larger shift observed here. The graphene has a much larger polarizability than h-BN. Hence, the strongly increased polarizability of the environment [40] compared with the isolated molecules in toluene solution could explain a large red-shift. Electronic interactions between conjugated π-electron systems of molecule and graphene (π-stacking), as evidenced by STM spectroscopy for non-alkylated PTCDI [28], may also play a role in changes of the optical bandgap. However, alkyl chains present here should reduce such interactions by maintaining the conjugated moiety at a larger distance from graphene. This is substantiated by the preservation of the absorption line-shape and the balance between vibronic contributions. This also means that the strong resonant molecule–molecule interaction evaluated above in free space is thus screened by the presence of the highly polarizable graphene substrate. These interpretations are consistent with the observation of a concentration of the oscillator strength for dense assemblies of PTCDA deposited on a dielectric substrate [18], in which case no significant spectral shift was observed. An exciting perspective could be given by hexagonal boron nitride (h-BN) monolayers, which combine a dielectric nature with an atomic-scale template similar to that of graphene [41].

## Conclusion

In conclusion, we have realised the self-assembly of alkylated PTCDI molecules onto a monolayer CVD graphene transferred on a transparent substrate. The molecules form a well-organized dense assembly, the parameters of which being accurately determined by STM. The polarized optical transmission spectra have been acquired at variable incidence thanks to the high optical transparency of the monolayer CVD graphene substrate. This confirms that the transition dipoles of adsorbed PTCDI are all oriented parallel to the substrate. The absorption is consistent with the measured density of molecules and presents mainly a rigid red-shift of the absorption line compared with the free molecules dispersed in solution. These changes are attributed to non-resonant interactions with the graphene layer and the neighbouring molecules.

## Experimental

The HOPG sample grade ZYB was purchased from SPI and the monolayer CVD graphene transferred onto transparent PET and fused silica samples were purchased from Megan-Technologies (Poland) and Graphenea (Spain), respectively. Both have been transferred from their copper CVD substrate using the standard PMMA technique [42]. The CVD graphene is polycrystalline, with typically 1 µm sized 2D domains. The PET/silica coverage by CVD graphene is ca. 95%.

*N*,*N*′-Ditridecylperylene-3,4,9,10-tetracarboxylic diimide (PTCDI-C13) was purchased from Sigma-Aldrich and used as received. It was dissolved in phenyloctane (99%, Chemos GmbH) for liquid–solid STM experiments or toluene (99.9%, Sigma-Aldrich) for monolayer depositions by drop casting or dip coating. For drop-casting experiments, the concentrations were adjusted so that the applied 5 µL droplet contains the quantity of molecules contained in a monolayer covering the entire 1 cm^2^ substrate, given the monolayer surface-density measured by STM (0.45 molecule per nm^2^). This target concentration evaluates to about 1.5 × 10^−5^ mol·L^−1^ and was adjusted with absorption spectroscopy applying the Beer–Lambert law with a molar absorptivity of ε = 87000 L·mol^−1^·cm^−1^ for various alkylated PTCDI [34]. For dip-coating experiments, the samples were immersed for 1 min in a toluene solution with the same concentration as for drop casting, and then gently rinsed with neat toluene for typically 5 min. The transferred monolayer CVD graphene remained unaltered even after prolonged immersion in toluene. Hence only the upper side of the graphene is exposed to the PTCDI solution.

The STM images were recorded under ambient conditions (ca. 300 K) with a custom-made digital system by the immersion of a 250 μm mechanically cut tip of Pt/Ir (90/10) purchased from Goodfellow into a 5 μL droplet of solution. The scanning piezoelectric ceramic was calibrated by means of atomic resolution obtained on HOPG images in *XY*-directions and with flame-annealed gold through the height of steps in the *Z*-direction. All the images were obtained at a quasi-constant current, i.e., in the variable-height mode. The images in Figure 1a,b were corrected for the thermal drift by combining two successive images with downward and upward slow-scan directions.

Optical absorption spectra at normal incidence were obtained with a Perkin-Elmer Lambda 650 spectrometer. Optical microspectroscopy was adapted on an Olympus IX71 microscope equipped with an Ocean Optics spectrometer QE-Pro. Variable incidence measurements were acquired on a custom-made goniometer bench using the same spectrometer, by monitoring the absorption at its maximum, at λ = 560 nm.
